# Multidimensional Benefits of a Tailored Exercise Program in Preventing Frailty: A Community-Based Approach

**DOI:** 10.3390/healthcare12212183

**Published:** 2024-11-01

**Authors:** Akihiro Kakuda, Yuko Sawada, Rika Okumura, Hiroshi Kinoshita, Tokie Anme

**Affiliations:** 1 Department of Physical Therapy, Morinomiya University Medical Sciences, 1-26-16, Nanko-kita, Suminoe-ku, Osaka 559-8611, Japan; 2 Graduate school of Health Science, Morinomiya University Medical Sciences, 1-26-16, Nanko-kita, Suminoe-ku, Osaka 559-8611, Japan; 3 Department of Public Welfare, Tobishima 490-1434, Japan; 4 Faculty of Medicine, University of Tsukuba 1-1-1 Tennodai, Tsukuba 305-8575, Japan

**Keywords:** frailty, community-based intervention, exercise program, older adults, Japan

## Abstract

Background/Objective: Frailty is a significant health concern in the aging population, particularly in Japan’s super-aging society. Community-based interventions show promise in frailty prevention; however, their effectiveness requires further investigation. This study aimed to evaluate the impact of a continuous municipal rehabilitation program on frailty status and physical function in older adults living in suburban Japan. Methods: This prospective observational study included 52 participants aged ≥ 65 years (13 males and 39 females) who underwent assessments at baseline and after six months. Participants were divided into Pre-old (65–74 years) and Older (≥75 years) groups. Frailty was assessed using the Kihon checklist (KCL), and physical function was evaluated using the New Physical Fitness Test. Changes in frailty status, physical function, and KCL subcategories were analyzed. Results: Frailty prevalence decreased significantly from baseline to 6 months (21.2% to 7.7%, *p* = 0.018). In the Pre-old group, significant improvements were observed in the sit-up (*p* = 0.035) and six-minute walking (*p* = 0.017) scores. The Older group showed significant improvements in KCL lifestyle (*p* = 0.023) and physical function (*p* = 0.018). Seven of ten initially frailty participants transitioned to a non-frailty status after 6 months. Conclusions: The Co-Creative Well-being System was associated with a reduction in frailty prevalence and improvements in physical function, with age-specific benefits observed. This community-based approach presents a promising strategy for addressing frailty in aging populations.

## 1. Introduction

Frailty is a multidimensional syndrome characterized by declines in physical, mental, and social function, often leading to increased vulnerability in older adults. The primary causes of frailty include inflammation, age-related sarcopenia, and changes in metabolic and immune functions [1,2]. Social isolation and cognitive decline further exacerbate health challenges, particularly in aging populations [3,4]. Early identification and targeted interventions are crucial to prevent its progression. Japan, as a super-aging society, faces significant public health challenges related to frailty, with 29.1% of the population aged 65 years or older [5]. The socioeconomic implications of frailty are profound, leading to shortened healthy life expectancy and increased medical and nursing care costs [6,7]. Therefore, frailty prevention and management are critical.

Effective interventions to mitigate frailty include exercise, social participation, and social support [8,9]. Regular physical activity improves strength and prevents functional decline, while social interaction reduces cognitive deterioration and delays the need for long-term care [10,11]. A holistic approach that integrates these interventions can yield synergistic effects, improving both physical and mental outcomes in frail individuals [12]. Despite these advances, community-based interventions often face challenges in information sharing across sectors. Access to individuals’ medical histories is frequently fragmented, limiting the ability to create personalized interventions. This highlights the need for a comprehensive, subject-centered information-sharing system.

**Figure 1:**
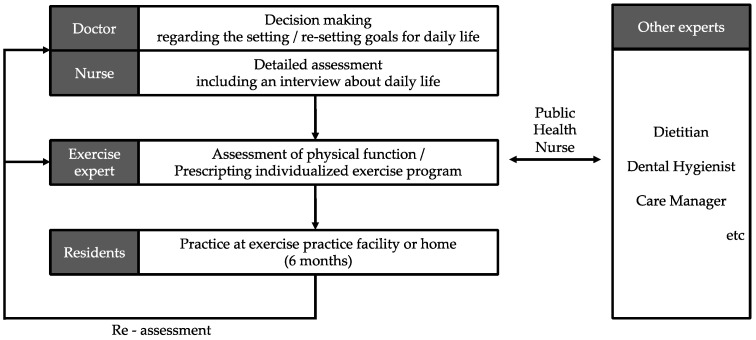
Conceptual diagram of the Tobi-Reha System.

**Figure 2:**
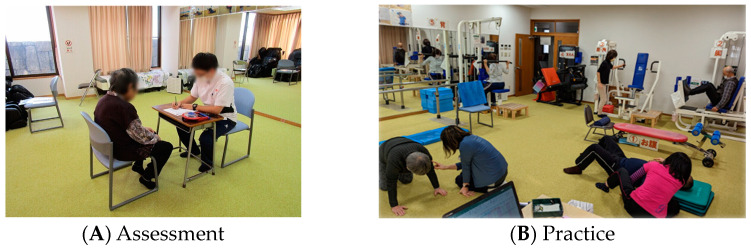
A view of the exercise practice facility.

The Co-Creative Well-being System, known as the “Tobi-Reha System” among residents, was developed in Tobishima Village, Aichi Prefecture, to address these challenges. This suburban area is characterized by a population structure that closely resembles that of Japan as a whole, making it an ideal location for community-based frailty prevention efforts. This municipal rehabilitation initiative emphasizes seamless communication between healthcare, nursing, and community services, fostering holistic well-being through social action and active resident participation (Figure 1). At the core of the Tobi-Reha System is a specialized exercise practice facility where experts (physiotherapists, health fitness programmers, public health nurses, etc.) assess physical function and provide individualized exercise programs. Residents receive an evaluation of their physical functions and advice on recommended exercises based on this evaluation, and they use the exercise practice facility according to their circumstances. This facility offers multi-component exercises, tailored to individual needs, and is equipped with various strength-building apparatuses (Figure 2). The exercise practice facility is positioned as an exercise promotion center that all residents can use at their own pace. The system encourages voluntary participation, promoting not only physical health but also social engagement, which is essential for comprehensive frailty prevention.

The aim of this study is to evaluate the effectiveness of the Co-Creative Well-being System, specifically the exercise practice facility, in reducing frailty and improving physical function in older adults. Additionally, this study seeks to identify opportunities for system improvement to better support the aging population.

## 2. Materials and Methods

### 2.1. Study Design and Participants

This study was conducted as part of the “community empowerment and care for well-being and healthy longevity (CEC) study,” a cohort project initiated in 1991. The CEC study involved approximately 4800 residents from Tobishima Village, Aichi Prefecture, a suburban area with a population structure similar to that of Japan as a whole. The present study employed a prospective observational design with a 6-month follow-up period.

The study population comprised individuals who underwent assessment in an exercise practice facility between 2019 and 2021 (n = 173,297 total visits). From this cohort, we selected 52 participants who met the following criteria: (1) aged 65 years or older at the time of initial evaluation and (2) available for follow-up assessment after a 6-month interval. The participants were divided into two age groups: 65–74 years (Pre-old group) and 75 years or older (Older group). This classification is commonly used in geriatric research because individuals aged 75 years and older are more likely to experience accelerated declines in physical function, cognitive abilities, and overall frailty compared to those in the 65–74 age group [13]. In cases where individuals resumed participation following a COVID-19-related interruption, their post-pandemic re-entry evaluation was considered the initial assessment for this study. The exclusion criteria included severe cognitive impairment and acute illness.

Table 1 shows the anthropometric data at the time of initial assessment in each age group. The ratio of males to females in each group was 8 males to 19 females in the Pre-old group and 5 males to 20 females in the Older group. There were no significant differences in the ratio of males to females between the groups.

### 2.2. Measures and Procedures

In addition to anthropometric measurements, physical function was assessed using the New Physical Fitness Test [14,15] and a questionnaire-based assessment using the Kihon checklist (KCL) [16].

The KCL is a widely used screening tool in Japan that evaluates frailty through a 25-item checklist across seven domains: lifestyle, physical function, nutritional status, oral function, houseboundness, cognitive function, and depression. The frailty group was defined as scoring 4 or more points for the total score of 25 items (classified as pre-frailty, frailty), and the non-frailty group scored 3 or fewer points (classified as robust) [16].

Physical function was evaluated using six items from the New Physical Fitness Test: grip strength, sit-ups, sit and reach, single-leg balance with eyes closed, a 10 m walk test with obstacles, and six-minute walking. Each item was scored on a 10-point scale, and an overall score was obtained by summing the six items. Grip strength was measured using a Smedley grip strength dynamometer (TKK5401; Takei Kikai Kogyo, Niigata, Japan), with the average of two maximal effort tests for each hand being calculated. Standardized equipment and procedures were followed for all tests based on the guidelines provided by the Ministry of Education, Culture, Sports, Science, and Technology in Japan [14,15].

The primary outcome measures were changes in frailty status and physical function scores over the 6-month period. Secondary outcomes included changes in the KCL subcategories.

### 2.3. Statistical Analysis

Frailty status was compared between the initial and follow-up assessments. For continuous variables, paired *t*-tests or Mann–Whitney U-tests were used, depending on the normality of the data. Discrete variables were compared using McNemar’s test. Between-group comparisons (Pre-old vs. Older) were conducted using Student’s *t*-test or the χ^2^ test for continuous and categorical variables, respectively. Statistical significance was set at *p* < 0.05. All statistical analyses were performed using SPSS (version 27.0; IBM).

### 2.4. Ethical Considerations

This study was approved by the Ethics Committee of Morinomiya University of Medical Sciences (approval number 2018-054). This study was conducted following the principles outlined in the Declaration of Helsinki. Written informed consent was waived as the analysis used anonymized data, and participants were allowed to opt out of this study in accordance with the Ethical Guidelines for Medical and Health Research Involving Human Subjects in Japan.

## 3. Results

### 3.1. Changes in Frailty Status over the Assessment Period

Frailty status at the initial and second evaluations is shown in Table 2. Among the forty-one participants classified as the non-frailty group at the first evaluation, one moved into the frailty group at the second evaluation. Conversely, of the ten participants initially classified as frailty, seven moved into the non-frail group after six months. This indicates a significant overall improvement in frailty status. The prevalence of frailty at baseline was 14.3% in the Pre-old group (65–74 years) and 28.0% in the Older group (≥75 years). The change in frailty prevalence was statistically significant in the Older group (*p* = 0.031) but not in the Pre-old group (*p* = 0.250).

### 3.2. Physical Function and KCL Scores

The baseline comparison of physical function between age groups is presented in Table 3. Grip strength and single-leg balance with eyes closed were higher in the Pre-old group (65–74 years), with the latter being statistically significant (*p* = 0.034). There was a trend toward higher scores in the Pre-old group for other physical fitness measures, but these differences did not reach statistical significance.

Table 4 shows the baseline frailty measured by the KCL, comparing the Pre-old and Older groups. While the Older group had higher average scores in the lifestyle and physical function domains, indicating greater frailty risks, these differences were not statistically significant at baseline.

### 3.3. Changes in Physical Function Measured by the New Physical Fitness Test

Table 5 presents changes in physical fitness test scores between the first and second evaluations. In the Pre-old group, significant improvements were observed in sit-ups (*p* = 0.010) and six-minute walking (*p* = 0.017). In the Older group, however, no significant changes were seen in the physical fitness tests.

### 3.4. Changes in Frailty Measured by the KCL

Changes in KCL scores are shown in Table 6. In the Older group, significant improvements were observed in the lifestyle (*p* = 0.040) and physical function (*p* = 0.034) categories, indicating improvements in frailty status and daily living capabilities. However, no significant changes were observed in the Pre-old group in any KCL subcategory. Only the Older group (≥75 years) improved in overall KCL score, driven by significant improvements in the lifestyle and physical function subcategories.

## 4. Discussion

Changes from the initial assessment to the reassessment 6 months later were analyzed to assess the impact of the Co-Creative Well-being System’s exercise practice facility on frailty prevention and improvement. The results demonstrate that the system not only contributed to frailty prevention but also actively reversed frailty in a significant proportion of participants. Specifically, 70% of those initially classified as frail moved to the non-frail group, with a particularly high rate of transition from frailty to non-frailty observed in the Older group. This significant reduction in frailty underscores the effectiveness of this community-based intervention.

The prevalence rates of frailty in the Pre-old and Older groups in the present study were 14.3% and 28.0%, respectively. The prevalence of frailty in the Japanese population aged 65 years or older was 12.5% [17] or 4.6–9.5% [18], which was higher than the results of this study. The prevalence of frailty among older people in Japan has increased from 11.5％ (2017) to 17.4% (2021) since the COVID-19 pandemic [19]. The study period was from 2019 to 2021, during which social isolation due to the COVID-19 pandemic likely increased the prevalence of frailty. Additionally, the Co-Creative Well-being System may have served as an important source of social interaction, potentially amplifying its impact on frailty prevention. Given that social isolation exacerbates frailty, it is possible that participants particularly benefited from the psychosocial aspects of the program, which may have been their only significant social engagement during the lockdown period.

It is also known that the prevalence of frailty generally increases with age [17,20], a trend similar to the results obtained in this study. The increase in the prevalence of frailty with age may be attributed to an increase in physiological decline, including age-related muscle weakness (sarcopenia), cognitive changes, and the accumulation of chronic diseases. The KCL used in this study has been shown to be a valid phenotypic assessment tool for frailty in a previous study [21]. The KCL is a comprehensive tool that can assess not only physical function but also cognitive function and social aspects, making it a suitable assessment method for the characteristics of older people in Japan. These results suggest that a multifaceted approach that considers complex health factors in addition to age is needed for the prevention and management of frailty.

### 4.1. Improvement in Frailty Status and Effectiveness of the System

The significant improvement in frailty status, especially in the Older group, suggests that the Co-Creative Well-being System is effective not only in preventing frailty but in reversing it. Previous studies report that around 31.4% of frail individuals move from frailty to non-frailty with multi-component exercises [9], whereas our study observed a much higher transition rate, further highlighting the effectiveness of the system. This suggests that the integration of personalized exercise guidance, community participation, and social interaction may provide a synergistic effect, improving outcomes beyond physical exercise alone.

### 4.2. Gender and Age Differences in Outcomes

It is important to note that a greater number of women participated in this system across both age groups. While previous research has highlighted gender differences in social participation and exercise adherence [22], there were no significant differences in the ratio of males to females between the age groups in this study, suggesting that the effects of the system are not influenced by gender.

The different results for the Pre-old and Older groups can be explained by the multifaceted nature of frailty, which includes physical, social, cognitive, and psychological factors. The Pre-old group showed significant improvements in physical function (e.g., sit-ups and 6 min walk test, etc.). While the Older group did not show any statistically significant improvements in physical function, they showed improvements in frailty status, as measured by the KCL subcategories (lifestyle and physical function, etc.). This suggests that for older participants, the program may have had a stronger impact on the non-physical aspects of frailty, such as cognitive and social health, which are equally important aspects of overall frailty improvement. The younger, physically fit, Pre-old group may have responded more quickly to the exercise component of the program. This indicates that while physical function may be more difficult to improve in older individuals, the decline in physical function can still be prevented through targeted exercise interventions [23,24].

### 4.3. Potential Mechanisms Behind the System’s Success

Several factors may account for the system’s success in preventing and improving frailty. First, the system is centered on voluntary participation, which may enhance participant motivation and long-term adherence. Additionally, the personalized exercise guidance provided by specialists ensures that participants engage in exercises appropriate for their individual capabilities, likely contributing to the improvements seen. Furthermore, the social interactions that naturally occur in the exercise practice facility likely provide additional psychosocial benefits, which are crucial for frailty prevention [12].

Multidimensional interventions, particularly those that address psychosocial factors in addition to physical function, are known to be highly effective in improving frailty [12]. The Co-Creative Well-being System appears to embody this multidimensional approach, as it encourages social interaction alongside physical activity, resulting in more comprehensive improvements in frailty status.

### 4.4. Future Directions

Future research should focus on several key areas to build on the findings of this study. First, investigating the long-term effects of the Co-Creative Well-being System through extended follow-up periods would provide valuable insights into its sustained impact on frailty prevention and improvement. Second, exploring the optimal frequency and duration of facility use could help develop more targeted recommendations for participants. Additionally, examining the specific components of the program that contributed most significantly to frailty improvement would allow further refinement of this intervention. Furthermore, investigating the factors that contribute to the transition from frail to robust status could provide crucial insights for developing more effective interventions. Finally, conducting cost-effectiveness analyses is crucial for assessing the feasibility of implementing similar programs in other communities.

The Co-Creative Well-being System represents a novel community-based approach to frailty prevention and improvement that integrates personalized exercise guidance with social interaction. Although our study has limitations, it provides a foundation for future research. It highlights the potential of such comprehensive, locally driven initiatives to address the complex challenges of frailty in aging populations.

### 4.5. Study Limitations

This study has several limitations. First, only those who were able to re-evaluate 6 months after the first use of the Co-Creative Well-being System were included, and there was a possibility of selection bias. Second, since the content of instruction for users and the frequency of use of the exercise practice facility were not examined in this study, more effective instruction content and recommended frequency of use should be considered in the future. Additionally, this study did not examine the content of the exercise guidance provided to participants or the frequency of facility use, which may have influenced the results. Furthermore, we did not collect data on potential confounding factors, such as comorbidities, medication use, or lifestyle changes outside this system, which could have influenced the results.

## 5. Conclusions

This study evaluated the effectiveness of the Co-Creative Well-being System, demonstrating its potential to not only maintain but also improve frailty status among elderly participants. Despite limitations, such as sample size and follow-up duration, the approach—which integrates personalized exercise with social interaction—promises significant benefits in frailty management. Further research is necessary to explore long-term effects and scalability, offering valuable insights for aging populations globally.

## Figures and Tables

**Table 1:** Baseline characteristics.

	Pre-Old Group (n = 27)			Older Group (n = 25)		
Age (Year)	70.4	±	2.7	81.0	±	4.5
Sex (Male/Female) (n)	8	/	19	5	/	20
Frailty Status (Frailty/non-Frailty) (n)	3	/	24	7	/	18
Standing Height (cm)	156.5	±	8.5	151.8	±	9.4
Body Weight (kg)	56.3	±	9.2	53.4	±	9.2
Body Mass Index (kg/m^2^)	22.8	±	2.6	23.1	±	2.9
Calf Circumference (Rt) (cm)	33.8	±	2.5	32.5	±	2.9
Calf Circumference (Lt) (cm)	33.7	±	2.3	32.8	±	2.9

Data are presented as mean ± SD, Pre-old group: 65–74 years old, Older group: over 75 years old.

**Table 2:** Frailty status measured by the Kihon checklist (KCL).

	1st Time			2nd Time			*p*-Value
	Non-Frailty Group	/	Frailty Group	Non-Frailty Group	/	Frailty Group	
All (n)	41	/	11	48	/	4	0.018
Pre-old group (n)	23	/	4	25	/	2	0.250
Older group (n)	18	/	7	23	/	2	0.031

Frailty group: classified as pre-frailty and frailty, non-frailty group: classified as robust, Pre-old group: 65–74 years old, Older group: over 75 years old.

**Table 3:** Baseline scores of the physical fitness test.

	Pre-Old Group (n = 27)			Older Group (n = 25)			*p*-Value
Total	29.7	±	9.0	24.2	±	9.7	0.038
Grip strength	5.4	±	1.6	4.6	±	1.7	0.073
Sit-ups	3.4	±	2.8	3.0	±	2.9	0.504
Sit and reach	5.6	±	2.0	5.2	±	2.8	0.495
Single-leg balance with eyes closed	7.2	±	3.3	5.5	±	3.1	0.034
10 m walk test with obstacles	4.8	±	2.4	3.6	±	2.2	0.051
Six-minute walking	3.2	±	1.7	2.3	±	1.5	0.074

Data are presented as average ± standard deviation, Pre-old group: 65–74 years old, Older group: over 75 years old.

**Table 4:** Baseline values of the Kihon checklist.

	Pre-Old Group (n = 27)			Older Group (n = 25)			*p*-Value
Total	1.30	±	2.80	2.44	±	3.37	0.188
Lifestyle	1.30	±	2.80	2.04	±	2.46	0.073
Physical Function	0.67	±	1.24	1.32	±	1.60	0.088
Nutritional Status	0.11	±	0.42	0.08	±	0.28	0.968
Oral Function	0.07	±	0.27	0.08	±	0.28	0.937
Houseboundness	0.00	±	0.00	0.08	±	0.28	0.138
Cognitive Function	0.00	±	0.00	0.12	±	0.44	0.138
Depression	0.00	±	0.00	0.40	±	1.80	0.138

Data are presented as average ± standard deviation, Pre-old group: 65–74 years old, Older group: over 75 years old.

**Table 5:** Changes in physical fitness test scores.

	1st Time			2nd Time			*p*-Value
Total							
65–74 years-old	29.7	±	9.0	31.0	±	9.2	0.199
≥75 years-old	24.2	±	9.7	24.6	±	10.0	0.693
Grip Strength							
65–74 years-old	5.4	±	1.6	5.3	±	1.6	0.327
≥75 years-old	4.6	±	1.7	4.7	±	1.5	0.450
Sit-ups							
65–74years-old	3.4	±	2.8	4.2	±	3.0	0.010
≥75 years-old	3.0	±	2.9	3.3	±	2.8	0.354
Sit and Reach							
65–74 years-old	5.6	±	2.0	5.6	±	2.1	0.917
≥75 years-old	5.2	±	2.8	4.8	±	2.5	0.107
Single-Leg Balance with Eyes Closed							
65–74 years-old	7.2	±	3.3	7.2	±	3.1	0.721
≥75 years-old	5.5	±	3.1	5.0	±	2.7	0.130
10 m Walk Test with Obstacles							
65–74 years-old	4.8	±	2.4	5.0	±	2.1	0.528
≥75 years-old	5.6	±	2.2	3.6	±	1.9	0.465
Six-Minute Walking							
65–74 years-old	3.2	±	1.7	3.8	±	1.8	0.017
≥75 years-old	2.3	±	1.5	2.5	±	1.3	0.465

Data are presented as average ± standard deviation.

**Table 6:** Changes in frailty measured by Kihon checklist values.

	1st Time			2nd Time			*p*-Value
Total							
65–74 years-old	1.30	±	2.80	0.67	±	1.52	0.165
≥75 years-old	2.24	±	3.37	1.16	±	1.60	0.018
Lifestyle							
65–74 years-old	1.30	±	2.80	0.67	±	1.52	0.165
≥75 years-old	2.04	±	2.46	1.16	±	1.60	0.040
Physical function							
65–74 years-old	0.67	±	1.24	0.41	±	1.01	0.228
≥75 years-old	1.32	±	1.60	0.76	±	1.09	0.034
Nutritional status							
65–74 years-old	0.11	±	0.42	0.04	±	0.19	0.157
≥75 years-old	0.08	±	0.28	0.08	±	0.28	1
Oral function							
65–74 years-old	0.07	±	0.27	0.11	±	0.32	0.564
≥75 years-old	0.08	±	0.28	0.04	±	0.20	0.564
Houseboundness							
65–74 years-old	0.00	±	0.00	0.04	±	0.19	0.317
≥75 years-old	0.08	±	0.28	0.00	±	0.00	0.157
Cognitive function							
65–74 years-old	0.00	±	0.00	0.00	±	0.00	1
≥75 years-old	0.12	±	0.44	0.08	±	0.40	0.317
Depression							
65–74 years-old	0.00	±	0.00	0.00	±	0.00	1
≥75 years-old	0.40	±	1.80	0.00	±	0.000	0.180

Data are presented as average ± standard deviation.

## Data Availability

The data presented in this study are not available due to privacy.
